# Transcytosis Involvement in Transport System and Endothelial Permeability of Vascular Leakage during Dengue Virus Infection

**DOI:** 10.3390/v10020069

**Published:** 2018-02-08

**Authors:** Chanettee Chanthick, Aroonroong Suttitheptumrong, Nantapon Rawarak, Sa-nga Pattanakitsakul

**Affiliations:** 1 Kasetsart University Veterinary Teaching Hospital, Faculty of Veterinay Medicine, Kasetsart University, Bangkok 10900, Thailand; lchanette@gmail.com; 2 Division of Molecular Medicine, Office for Research and Development, Faculty of Medicine Siriraj Hospital, Mahidol University, Bangkok 10700, Thailand; n_1103@hotmail.com; 3 Division of Molecular Medicine, Office for Research and Development, 4th Floor SiMR Building, 2 Wanglang Road, Faculty of Medicine Siriraj Hospital, Bangkoknoi, Bangkok 10700, Thailand

**Keywords:** Thailand, transcytosis, transport system, endothelial permeability, vascular leakage, dengue virus infection

## Abstract

The major role of endothelial cells is to maintain homeostasis of vascular permeability and to preserve the integrity of vascular vessels to prevent fluid leakage. Properly functioning endothelial cells promote physiological balance and stability for blood circulation and fluid components. A monolayer of endothelial cells has the ability to regulate paracellular and transcellular pathways for transport proteins, solutes, and fluid. In addition to the paracellular pathway, the transcellular pathway is another route of endothelial permeability that mediates vascular permeability under physiologic conditions. The transcellular pathway was found to be associated with an assortment of disease pathogeneses. The clinical manifestation of severe dengue infection in humans is vascular leakage and hemorrhagic diatheses. This review explores and describes the transcellular pathway, which is an alternate route of vascular permeability during dengue infection that corresponds with the pathologic finding of intact tight junction. This pathway may be the route of albumin transport that causes endothelial dysfunction during dengue virus infection.

## 1. Clinical Manifestations of Dengue Virus Infection

Dengue virus (DENV) infection can produce a wide variety of illnesses. Most dengue infections are asymptomatic; however, a minority of cases show clinical signs that range from undifferentiated fever, to mild form of dengue infection (dengue fever (DF)), to severe form of dengue infection (dengue hemorrhagic fever (DHF)) [1]. Undifferentiated fever that presents as a simple fever and that is indistinguishable from fevers caused by other viral infections usually follows a primary dengue infection. DF occurs more frequently in older children, adolescents, and adults, with DHF more commonly occurring in children less than 15 years of age that have sustained repeated dengue infection [2]. Both DF and DHF are characterized by a sometimes biphasic 2–7 day persistently high fever (39–41 °C). Hemorrhagic tendencies, thrombocytopenia, and hepatomegaly are usually observed in both DF and DHF [3,4]. The hallmark of DHF that distinguishes it from DF is the presence of varying degrees of plasma leakage that can develop into hypovolemic shock and circulatory failure [5]. This condition is referred to as dengue shock syndrome (DSS), and it generally occurs when the patient’s body temperature drops to 37.5–38 °C or less around the time of defervescence, which is usually on day 3–7 of illness. DSS is clinically associated with hemoconcentration, which is defined as reduced plasma volume and increased concentration of circulating red blood cells. Patients with DSS may exhibit abnormal signs, such as low blood pressure (<20 mmHg), change in pulse rate, delayed capillary refill time (>3 s), cold clammy skin, restlessness, and abdominal pain [4]. Shock is reversible if prompt and adequate fluid therapy is given. Without treatment, the patient may die within 12 to 24 h [2]. Patients with prolonged shock tend to develop electrolyte imbalance, multi-organ failure, and severe bleeding from various organs—all of which portend poor prognosis and high mortality. The World Health Organization (WHO) recently revised their classification of dengue into non-severe dengue with/without warning signs and severe dengue [6]. In non-severe dengue, the new system provides signs and symptoms that clinicians should observe for that occur in patients before deterioration of conditions. These warning signs facilitate early detection of high-risk dengue patients and can be used as a guideline for clinical monitoring and therapy [6].

## 2. Immunopathogenesis of DHF/DSS

The pathogenesis of dengue virus infection remains inconclusive and is still being widely debated. The mechanism of DHF/DSS is a complex interplay of viral and host factors, with several hypotheses having been proposed based on clinical and epidemiological observations [7]. The incidence of DHF/DSS peaked in two populations of young children [8]. The first peak occurred in first-time infected infants born to dengue-immune mothers. The infants acquired maternal dengue antibodies across the placenta. DHF/DSS developed in this group during a time of decreased maternal immunity or after exposure to different serotypes from the infected mother. The second peak occurred in young children who had experienced an earlier mild or subclinical dengue infection, and who were later infected with a different dengue serotype. These two observations generated interest in the possibility that an enhancing antibody is involved in the pathogenesis of DHF/DSS. Patients that develop primary dengue virus infection are usually asymptomatic and will generate immunity to homologous strains of the virus, which results in lifelong protection against that particular serotype [9,10]. In a secondary dengue infection–non-neutralizing antibodies, cross-reactive antibodies, antibodies generated from a previous dengue infection or that were acquired from maternal immunity, and subneutralizing homologous antibodies recognize dengue epitopes, but they cannot neutralize the virus. Instead, they facilitate entry of the virus into mononuclear phagocytic cells, which results in virus burden and increased risk of developing DHF/DSS. Antibody-dependent enhancement (ADE) is usually found more in patients with secondary dengue infection than in patients with a primary infection [11]. Viral burden in dengue virus infection has been suggested as a factor that increases disease severity [12,13,14]. Patients who developed DHF/DSS had a peak virus titer that was 100-fold to 1000-fold higher than those who developed DF [12]. During the period when patients were affected by plasma leakage, a low viral load was observed in circulation. Host immunologic response is supposed to play a key role in mediating increased vascular permeability. While the mechanism remains unclear, several immunologic constituents are suspected of participating in the pathogenesis, including T lymphocytes and the complement system. It has been suggested that CD4^+^ and CD8^+^ T lymphocytes are activated by infected monocytes/macrophages and dendritic cells, which results in increased production of many cytokines and chemokines [15]. Original antigenic sin has been hypothesized to be the key mechanism. Secondary infection with different dengue serotypes stimulates extensive proliferation of cross-reactive, low-affinity memory T-cells specific to the previous infection. The activation, proliferation, and death of these low-affinity cells lead to ineffective virus clearance and massive cytokine release [16]. The plasma level of various cytokines and chemokines was found to be significantly higher in DHF/DSS patients (e.g., interleukin (IL)-2, IL-6, IL-8, IL-10, IL-12, and IL-18; transforming growth factor (TGF)-β1; interferon (IFN)-γ; and, tumor necrosis factor (TNF)-α) [17,18,19]. These mediators may contribute to endothelial cell damage and/or transient compromise of endothelial barrier function.

Relative to the complement system, in vitro studies revealed that dengue virus infection led to C3 activation and C5b-9 complex formation on the surface of endothelial cells [20,21]. Dengue NS1 protein triggered complement activation both on the cell-surface and during the fluid phase. The formation of C5b-9 complex might activate cellular reactions or cytokine production that adversely affect vascular permeability.

Other host factors for increased risk of developing DHF/DSS include female gender, younger age, good nutritional status, Caucasian or Mongoloid race, and human leukocyte antigen (HLA) class I alleles [2,22]. In addition virus factors, certain dengue strains are considered to be another risk factor for more severe disease [23]. Southeast Asian genotype of DENV2 and DENV3 are frequently associated with severe disease in secondary dengue infection [24,25], whereas American genotype of DENV2 was not found to cause DHF/DS [26].

## 3. Pathophysiology of Vascular Permeability

The vascular system has the vital function of supplying body tissues with nutrients and clearing tissues of metabolic waste products. Endothelium, as part of the vascular system, consists of a monolayer of endothelial cells that lines the interior layer of the entire vascular system. It acts as a semi-permeable barrier that regulates the exchange of molecules and fluids between blood circulation and body tissue compartments [27,28]. Endothelial cells are able to direct their paracellular (through the interendothelial junction) and transcellular (through the endothelial cell) pathways to facilitate transport of proteins, solutes, and fluid [27].

Plasma is a relatively clear, yellow-tinted liquid component of blood that accounts for approximately 55% of blood volume. It consists mostly of water, substances used to supply body tissue (i.e., electrolytes, hormones, nutrients, and oxygen), and metabolic waste discarded from tissues, like urea and carbon dioxide. Apart from the substances that are moved in and out of cells, plasma proteins are also essential constituents of plasma. Three major plasma proteins (i.e., albumin, globulin, and fibrinogen) function as colloid oncotic agents, which are essential for keeping fluid within blood vessels. Having a semi-permeable property, endothelium behaves like a molecular sieve with an average pore radius of 3 nm [27,29]. Gasses and small solutes are able to pass through endothelial cells by diffusion or through the interendothelial junction [27,30], whereas water traverses the endothelial barrier through both the interendothelial junction and transcellular water-transporting membrane channels known as the aquaporin [27]. Most plasma proteins are macromolecules that cannot pass through an intact interendothelial junction. Some proteins (i.e., albumin, low-density lipoproteins, and insulin) are able to be transported through endothelial cells via caveolae [27,31,32,33,34,35,36]. Of the three major proteins in serum, albumin is the most abundant, accounting for 54% of total plasma protein. Albumin is a highly charged protein with a molecular weight of 69 kDa that is newly synthesized by hepatocytes and secreted into blood circulation. The high net negative charge of albumin can attract Na^+^ cations and water to follow and move across the semi-permeable capillary membrane into blood circulation or into the extravascular compartment [37]. These properties highlight albumin as the largest contributor of colloid oncotic pressure, providing 75% (21.8 mmHg) of total plasma oncotic pressure (28 mmHg) [38]. In addition to having “chief oncotic agent” properties, albumin functions as a cargo chaperone that binds to many substances (e.g., hormones, fatty acids, metal, etc.) in plasma and facilitates their delivery across the endothelium [27].

Relative to vascular permeation under physiologic condition, basal vascular permeability (BVP) facilitates the supply of nutrients to surrounding tissues [39]. A large quantity of gas, water, and small solutes are allowed to freely exchange, but certain amount of certain plasma proteins (mainly albumin) is permitted to extravasate via the transcellular route. Albumin and some macromolecules are transported across the endothelial cell within discrete membrane-bound vesicles that are called caveolae. Caveolae comprise about 15% of total endothelial cell volume (10,000–30,000 caveolae/cell) [27,28,31]. Albumin transcytosis is initiated by the binding of albumin to the albumin-binding protein (gp60) at the apical (luminal) membrane of an endothelial cell [27,32,40,41]. After binding, both receptor-bound and fluid-phase albumins are internalized within caveolae [27,41], which is followed by scission and the release of the vesicle from the plasma membrane. Released caveolae move through the cytoplasm to the opposite cell surface where they fuse with the target membrane and release albumin to extravascular compartments by exocytosis, thereby causing a rapid flux of cation and water across the normal vessel [27,31]. The fluid passing from circulation into normal tissues under basal condition is a plasma filtrate that largely consists of water and small solutes but only a small amount of protein [30]. Albumin and plasma filtrate that leak across the endothelium are rapidly removed by the lymphatic system [27,31,32]. Amplification of albumin transcytosis within endothelial cells has also been suggested as a contributing factor in disease pathogenesis [6,34,42,43]. By way of example and in a setting of acute lung injury, an increase in caveolae-mediated transendothelial albumin permeability resulted in pulmonary edema or extravascular fluid accumulation in the lung [42].

Unlike basal vascular permeability (BVP), acute vascular hyperpermeability (AVH) and chronic vascular hyperpermeability (CVH) are the consequences of pathologic changes. Both are characterized by the dramatically increased vascular permeation. Acute vascular hyperpermeability (AVH) occurs in response to exposure to any of the vascular permeabilizing factors (e.g., histamine, vascular endothelial growth factor (VEGF), tumor necrosis factor (TNF)-α) that are involved with inflammation and infection [27,30]. After exposure, two independent mechanisms [destabilization of adherens junctions (the major component of endothelial cell-cell junction) and activation of actomyosin contractility within endothelial cells] are mediated via c-Src, Ca^2+^ signaling and RhoGTPase pathways. Internalization and disassembly of phosphorylated VE-cadherin and β-catenin results in adherens junction destabilization, whereas phosphorylation of myosin light-chain kinase (MLCK) leads to actomyosin contraction [44,45,46,47,48]. These mechanisms lead to the dynamic opening and closing of endothelial cell-cell junctions, which cause reversible endothelial cell contraction and the dissociation and reassembly of junctional complex proteins [27,29,30,31]. This results in an influx of plasma into tissues and/or extravascular spaces via the paracellular route due to disruption of the interendothelial junction. The extravasated fluid, an exudate that is rich in plasma proteins, approaches the level found in plasma [27,30,31]. Chronic vascular hyperpermeability (CVH) occurs during chronic exposure to vascular permeabilizing factors, which results in profound changes in vascular structure. CVH is commonly found in tumors, healing wounds, and chronic inflammatory diseases. The fluid that extravasates is an exudate that closely mirrors the overall composition of plasma [30].

## 4. Characteristics of Plasma Leakage in DHF/DSS

Plasma leakage in dengue virus infection is not a generalized type of edema; rather, it is mostly limited to the pericardial, pleural, and abdominal cavities. Pleural effusion is most common [3,49]. DHF patients normally start to show abnormal chest radiographic changes (i.e., small amount of fluid accumulation) on the first day of fever. Progressive changes occur during the first week and then improve during the second week [50]. The critical period of life-threatening hypotension secondary to plasma leakage is at the end of the febrile phase after defervescence [1]. Effusion is normally yellowish, watery, and occasionally blood-tinted. Effusion volume varied from 50 mL in a six-month-old infant to about 700 mL in a 12-year-old child [51,52]. For purposes of comparative description, 700 mL of fluid loss in a twelve-year-old child is estimated to be equal to one-fourth of the patient’s total blood volume. Compared to normal plasma protein concentration of 6.0–8.5 g/dL, the protein content of effusion is between 3.4–5.4 g/dL. The ratio between percentage of albumin in plasma and the percentage of albumin in effusion was 0.7–0.9, whereas the same ratio for globulins was 1.4–4.6, as determined by paper electrophoresis [51]. The duration of effusion is approximately 24–48 h [1]. After this effusion period in an uncomplicated case, the patient normally experiences a spontaneous and rapid recovery without sequelae [52].

## 5. Endothelial Cells Involved in Plasma Leakage during Dengue Virus Infection

Endothelium, as the site of plasma leakage in dengue virus infection, has been investigated in several experimental models. Cytokines and chemokines have been suggested as important factors that play a role in dengue infection and that influence severity in dengue patients [18,19]. The endothelial cells that were the main targets of investigation were induced by several mediators secreted by immunologic components during infection, including monocytes, macrophages, dendritic cells, and T-lymphocytes [4,49]. Several proinflammatory mediators, including platelet-activating factor (PAF), macrophage migration inhibitory factor (MIF), matrix metalloproteinase (MMP)-9, and monocyte chemoattractant protein (MCP)-1, had significant effect on endothelial cells by reducing the expression of platelet and endothelial cell adhesion molecule (PECAM)-1 and vascular endothelial (VE)-cadherin on the endothelial membrane, by disrupting the distribution of tight junction ZO-1 protein, and by redistributing F-actin fiber [50,53,54,55]. Disruption of the interendothelial junction, and the subsequent increased vascular permeability was the predicted outcome of these mediator-endothelial cell interactions. Study of the synergistic effect of DENV and TNF-α to induce reorganization of adhesion molecules in human endothelial cells was recently described [56].

The dengue virus influenced infected endothelial cells to secrete a group of proinflammatory cytokines and chemokines that included interleukin (IL)-8, RANTES, MMP-2, and vascular endothelial growth factor (VEGF) [20,57,58,59]. Dengue infection caused suppression of TNF-α-mediated hyperpermeability in human umbilical vein endothelial cell (HUVEC) monolayer within 72 h after infection [60]. Peripheral blood mononuclear cells (PBMCs) also participated in the increase in permeability of dengue-infected endothelial cells by reducing cell-surface expression of VE-cadherin [61].

Glycocalyx, which consists of proteoglycan, glycoprotein, glycosaminoglycan, and absorbed plasma proteins, acts as the charge barrier that lines the luminal side of endothelium [43]. The functions of the glycocalyx include stabilization of endothelial cells and acting as a mechanotransducer of shear stress [62]. Shedding or loss of glycocalyx in pathological conditions (e.g., ischemia, infection, diabetes) leads to vascular hyperpermeability, leukocyte-endothelial interaction, vascular inflammation, and thrombosis [63]. This phenomenon has been proposed as another factors that plays a role in plasma leakage in dengue virus infection. Clinical study of serum hyaluronic acid, heparan sulfate, chondroitin sulfate, and syndecan-1, which were proteins shed from luminal surface in dengue patients, revealed increased levels of serum hyaluronic acid and heparan sulfate in dengue patients compared to non-dengue patients, while no difference was observed between patients with DF and DHF [64]. Another clinical study reported concurrently high levels of syndecan-1 and claudin-5, the latter of which is a constituent of tight junctions that has been associated with severe plasma leakage [65]. Moreover, some in vitro studies found that dengue NS1-induced disruption of sialic acid and heparan sulfate via the induction of intraendothelial sialidase and cathepsin L led to paracellular hyperpermeability [66,67].

Altered proteins following dengue virus infection of human endothelial (EA.hy926) cells were studied using high-throughput two-dimensional gel electrophoresis (2-DE) analysis, followed by quadrupole time-of-flight mass spectrometry (Q-TOF MS) and tandem mass spectrometry (MS/MS) [68]. Kanlaya et al. reported that 15 proteins involved in mRNA stability/processing, transcription and translation regulation, molecular chaperoning, oxidative stress response/regulation, cytoskeletal assembly, protein degradation, and cellular metabolism were altered in response to dengue virus infection [68]. That study focused on the actin cytoskeleton, which was upregulated 2.99 times after infection with DENV2 for 24 h. By indirect immunofluorescence staining, decreased expression and reorganization of actin cytoskeleton and junctional proteins, including VE-cadherin, tight junction ZO-1, and PECAM-1, were observed in the infected cells, whereas increased actin stress fiber was found in adjacent non-infected cells. The study of dengue virus infection in endothelial cells also showed alteration of actin cytoskeleton and junctional molecules. A subcellular proteomic study by Pattanakitsakul et al. revealed that 35 proteins were altered in dengue virus-infected EA.hy926 cells [69]. Upregulated proteins were mainly involved in the endocytosis system and cellular metabolism, while downregulated proteins were participating in molecular chaperoning. Among these proteins, albumin was 5.09 times upregulated in a cytosolic fraction of DENV2-infected EAhy.926 cells.

## 6. Transcytosis as an Alternative Transport System in Endothelial Cells

**Figure 1:**
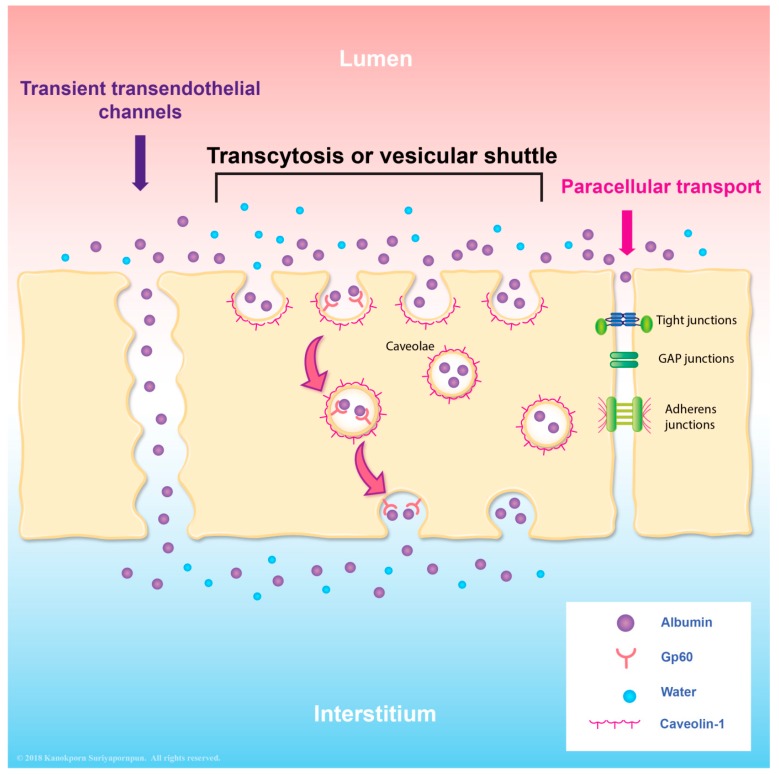
Illustration describing the vesicular transport of albumin. There are several transport pathways, including paracellular transport of molecules through the cell junction. Another pathway is transcytosis, which includes the transendothelial channels and vesicular shuttle. Transcytosis occurs via fusion-fission model or via binding between gp60 and albumin molecules which are mediated via caveolae.

According to histologic studies, the rare structural injury [51,70,71] of endothelial cells was detected in DHF/DSS autopsies. These findings emphasized that functional alteration of endothelial cells is likely to play an important role. As mentioned earlier, caveolae-mediated albumin transcytosis is the hallmark feature of endothelial cells. At basal level, albumin transcytosis is initiated by the binding of albumin to its receptor, a 60-kDa glycoprotein (gp60) located on the endothelial luminal surface [40,41]. After binding, gp60 clustering and its interaction with caveolin-1 (the primary structural protein of caveolae) activates Src tyrosine kinases (the key switch that phosphorylates caveolin-1) and the scission protein (dynamin-2), which causes the release of caveolae from the plasma membrane and stimulates transcellular transport of receptor-bound and fluid-phase albumin [27,32,35,39,72,73,74,75,76,77]. The resulting increased Src kinase activity, which is mediated by a group of pathologic and non-pathologic stimuli, including neutrophil-endothelial interaction [42], oxidant [78], isoflurane [79], atrial natriuretic peptide [80,81], and inflammatory mediators (thrombin, high mobility group box protein 1) [81,82], enhances caveolae-mediated transendothelial albumin transport that is involved in edema formation. Caveolae-mediated albumin transcytosis has also been shown to be increased in endothelial cells during dengue virus infection, although the molecular mechanism has not been elucidated [83] (Figure 1). In addition to paracellular hyperpermeablity, transcellular hyperpermeability may also play a role in the pathogenesis of plasma leakage in DHF/DSS patients [62].

Pathologic evaluation and electron microscopic study of dermal vessels in 60 dengue hemorrhagic fever (DHF) patients found most endothelial junctional complexes to be intact, interendothelial gaps present only in some cases, and prominent increase in pinocytotic vesicles in cytoplasm of endothelial cells [70,84]. Given that only a small amount of thoracocentesis data was available due to the short duration of leakage, pleural effusions from 38 DHF patients was analyzed. Those studies revealed all of these effusion specimens not to be protein-rich exudates, which is commonly seen in many infectious diseases, but exudates with low amount of protein and cells that were classified by Light’s criteria as transudate [50,85,86]. A larger proportion of albumin in effusion than in plasma indicated selective extravasation [51,86]. In the setting of vascular physiology, transudative effusion is a plasma filtrate that is caused by an imbalance between hydrostatic and oncotic pressure across the intact endothelium [87,88]. In contrast to exudative effusion, the selective extravasation of albumin and plasma filtrate in transudate causes a lack of tissue factor activation and coagulation [89,90], which is the process that plays a role in the development of pleural and/or peritoneal inflammation. Without the complications of inflammation and adhesion, transudative effusion can be entirely removed via capillary reabsorption and lymphatic drainage. This characteristic of transudate is strongly correlated with uneventful recovery with no sequelae of effusion that can be observed in DHF/DSS patients.

## 7. Conclusions

Transcellular endothelial permeability is the functional pathway that mediates vascular permeability under physiologic condition. This so-called basal vascular permeability (BVP) is the crucial function of endothelial cells. This pathway is associated with several disease pathogeneses, and pathologic findings in DHF/DSS patients suggest that transcellular endothelial hyperpermeability may be implicated in plasma leakage during dengue infection. Transendothelial hyperpermeability is recommended for further study of plasma leakage in dengue virus infection.
